# Neuroendocrine and cardiac metabolic dysfunction and NLRP3 inflammasome activation in adipose tissue and pancreas following chronic spinal cord injury in the mouse

**DOI:** 10.1042/AN20130021

**Published:** 2013-09-04

**Authors:** Gregory E. Bigford, Valerie C. Bracchi-Ricard, Robert W. Keane, Mark S. Nash, John R. Bethea

**Affiliations:** *The Miami Project to Cure Paralysis, University of Miami Miller School of Medicine, Miami, FL, U.S.A.; †Department of Physiology, University of Miami Miller School of Medicine, Miami, FL, U.S.A; ‡Department of Neurological Surgery, University of Miami Miller School of Medicine, Miami, FL, U.S.A.; §Department of Rehabilitation Medicine, University of Miami Miller School of Medicine, Miami, FL, U.S.A.; ∥Department of Microbiology and Immunology, University of Miami Miller School of Medicine, Miami, FL, U.S.A.

**Keywords:** cardiovascular disease, metabolism, neuroendocrine, pathophysiology, signal transduction, spinal cord injury, ASC, apoptosis-associated speck-like protein containing a caspase recruitment domain (CARD, q.v.), ARC, arcuate nucleus, CNS, central nervous system, CVD, cardiovascular disease, DAMPS, damage–associated molecular pattern molecules, FAO, fatty acid oxidation, HRP, horseradish peroxidase, IACUC, Institutional Animal Care and Use Committee, IL, interleukin, JAK, Janus kinase, MYPT1, myosin phosphatase target subunit 1, NLRP3, NOD-like receptor family, pyrin domain containing 3, NPY, neuropeptide Y, PFA, paraformaldehyde, POMC, proopiomelanocortin, PVN, paraventricular nucleus, RBD, Rho-binding domain, ROCK, Rho-associated kinase, SCI, spinal cord injury, STAT, signal transducer and activator of transcription, VAT, visceral adipose tissue

## Abstract

CVD (cardiovascular disease) represents a leading cause of mortality in chronic SCI (spinal cord injury). Several component risk factors are observed in SCI; however, the underlying mechanisms that contribute to these risks have not been defined. Central and peripheral chronic inflammation is associated with metabolic dysfunction and CVD, including adipokine regulation of neuroendocrine and cardiac function and inflammatory processes initiated by the innate immune response. We use female C57 Bl/6 mice to examine neuroendocrine, cardiac, adipose and pancreatic signaling related to inflammation and metabolic dysfunction in response to experimentally induced chronic SCI. Using immuno-histochemical, -precipitation, and -blotting analysis, we show decreased POMC (proopiomelanocortin) and increased NPY (neuropeptide-Y) expression in the hypothalamic ARC (arcuate nucleus) and PVN (paraventricular nucleus), 1-month post-SCI. Long-form leptin receptor (Ob-Rb), JAK2 (Janus kinase)/STAT3 (signal transducer and activator of transcription 3)/p38 and RhoA/ROCK (Rho-associated kinase) signaling is significantly increased in the heart tissue post-SCI, and we observe the formation and activation of the NLRP3 (NOD-like receptor family, pyrin domain containing 3) inflammasome in VAT (visceral adipose tissue) and pancreas post-SCI. These data demonstrate neuroendocrine signaling peptide alterations, associated with central inflammation and metabolic dysfunction post-SCI, and provide evidence for the peripheral activation of signaling mechanisms involved in cardiac, VAT and pancreatic inflammation and metabolic dysfunction post-SCI. Further understanding of biological mechanisms contributing to SCI-related inflammatory processes and metabolic dysfunction associated with CVD pathology may help to direct therapeutic and rehabilitation countermeasures.

## INTRODUCTION

Extensive research affirms the prevalence of chronically acquired all-cause CVD (cardiovascular disease) and related neuroendocrine/metabolic disorders following traumatic SCI (spinal cord injury) (DeVivo et al., 1999; Garshick et al., 2005; Myers et al., 2007; Nash and Mendez, 2007; Wahman et al., 2011). The clustering of several component risk factors, described as the cardiometabolic syndrome, is observed in persons with SCI, including central obesity (Chen et al., 2006; Liang et al., 2007; Groah et al., 2009; Wahman et al., 2011), dyslipidemia (Brenes et al., 1986; Bauman et al., 1992; Maki et al., 1995), hypertension (in persons with paraplegia) (Nash and Mendez, 2007; Wahman et al., 2011), and either impaired fasting glucose or frank diabetes (Bauman and Spungen, 2001; Wahman et al., 2011). Adipose-derived peptide hormones, described as adipokines, contribute to both central and peripheral neuroendocrine regulation of energy metabolism (Ahima et al., 2006a, 2006b), and pathological dysregulation of their gene products and signal integration contribute to pro-inflammatory responses and metabolic dysfunction (Ouchi et al., 2011) associated with cardiometabolic risk (Deng and Scherer, 2010). Specifically, the adipokine leptin has been shown to play an integral role in regulating hypothalamic function (Zhang et al., 1994; Yu et al., 1997; Mantzoros, 1999) and obesity-linked metabolic and vascular diseases (Prins, 2002). Several studies have shown that subjects with SCI have significantly elevated serum leptin levels compared to control populations (Baumann et al., 1996; Huang et al., 2000; Wang et al., 2005) and that increased leptin levels in this population is strongly associated with visceral fat area (Jeon et al., 2003; Maimoun et al., 2004; Maruyama et al., 2008). Elevated serum leptin is also observed in rodent models of SCI (Gezici et al., 2009; Wang et al., 2011), and recent data, including our own, have determined significant alterations in leptin signaling following modeled CNS (central nervous system) injury. Notably, we show biological evidence for leptin resistance following chronic SCI (Bigford et al., 2012), an established mediary of hypothalamic inflammation (Ozcan et al., 2004; Hosoi et al., 2008; Zhang et al., 2008). However, the extent of central signal dysregulation remains unclear, and the relationship to peripheral inflammation associated with comorbidities of metabolic disorders associated with CVD progression and diabetes risk following SCI have yet to be explored.

Central leptin signaling in the hypothalamic ARC (arcuate nucleus) directly influences several components of the central melanocortin system, which is known to regulate metabolic energy balance (Coll et al., 2004; Cone, 2005). Leptin pathways regulate a number of neuropeptides, activating POMC (proopiomelanocortin) transcription, and, conversely, inhibiting NPY (neuropeptide Y), which normally suppresses the primary melanocortin system (Roseberry et al., 2004; Mercer et al., 2011). Leptin is also independently linked with HR (heart rate) (Carlyle et al., 2002; Ren, 2004), and stimulation of cardiac FAO (fatty acid oxidation) and oxygen consumption (Atkinson et al., 2002) via a JAK2/STAT3(signal transducer and activator of transcription 3)/p38 MAPK (mitogen-activated protein kinase) mechanism (Sharma et al., 2009). FAO is associated with inhibition of cardiac function and mechanical efficiency (Nickola et al., 2000; Wold et al., 2002; Sharma et al., 2009; Lopaschuk et al., 2010), and FA intermediates increase oxidative stress leading to activation of pro-apoptotic, -fibric, and -inflammatory pathways (Bielawska et al., 1997; Severson, 2004). Further, leptin-induced p38 MAPK nuclear import results in cardiomyocyte hypertrophy, involving the small GTPase RhoA and ROCK (Rho-associated kinase) (Zeidan et al., 2006; Zeidan et al., 2008), identifying an essential contribution of RhoA/ROCK to cardiac dysfunction. Importantly, studies have shown an association between leptin and several pro-inflammatory cytokines with endothelial dysfunction and atherosclerosis (Dubey and Hesong, 2006; Korda et al., 2008; Hou and Luo, 2011), supporting the involvement of this potent adipokine in global inflammatory processes associated with CVD progression.

Several other peripheral tissues represent bioactive sites of pro-inflammatory processes contributing to comorbid disease risk. Previous evidence demonstrates that both VAT (visceral adipose tissue) and pancreatic inflammation mediated by macrophage and T-cell activation of the NLRP3 (NOD-like receptor family, pyrin domain containing 3) inflammasome is principal in the induction of obesity, insulin resistance, and impaired glucose metabolism (Vandanmagsar et al., 2011; Wen et al., 2011; Goossens et al., 2012). Intracellular formation of the NLRP3 inflammasome incites the autocatalytic activation of caspase-1, and the subsequent activation and release of the pro-inflammatory cytokines IL-1β (interleukin-1 β) and IL-18 (interleukin 18) (Agostini et al., 2004), involved in acute response and chronic autoinflammatory disease (Dinarello, 2009; Masters et al., 2009; Troseid et al., 2010). Despite being implicated in metabolic dysfunction, whether these biological processes linked to cardiac metabolism, VAT and pancreatic inflammation are induced by SCI has yet to be explored.

In this study, we investigate inflammatory processes and metabolic pathophysiology related to CVD progression and diabetes risk following traumatic SCI. We provide evidence that hypothalamic signaling proteins are significantly altered following chronic SCI, and show dysregulation in cardiac metabolism. We further show the activation of pro-inflammatory signaling platforms associated with metabolic dysfunction in both VAT and pancreatic tissue. These findings demonstrate biological mechanisms related to cardiometabolic risk factors, and indicate a multi-system, chronic state of inflammation and maladaptive metabolism following SCI.

## MATERIALS AND METHODS

All animal protocols were approved by the University of Miami IACUC (Institutional Animal Care and Use Committee) and are in accordance with National Research Council guidelines for the care and use of laboratory animals.

### Traumatic SCI

Surgeries were performed at the Animal and Surgical Core Facility of the Miami Project to Cure Paralysis according to protocols approved by the IACUC of the University of Miami. Contusion injury was induced with the Infinite Horizon Impactor device adapted to the mouse. The infinite Horizon impactor device has been established in producing precise, graded contusion, with reproducible lesion volume and functional outcomes assessed using BBB (Basso, Beattie, Bresnahan) and BMS (Basso Mouse Scale) open-field locomotor rating scales (Nishi et al., 2007). In brief, mice were anesthetized with an intraperitoneal injection of ketamine (80–100 mg/kg) and xylazine (10 mg/kg). Complete anesthetization was determined by the lack of a stereotypical retraction of the hindpaw in response to a nociceptive stimulus. Mice were then subjected to a laminectomy at vertebrae T9 and the exposed spinal cord was injured at a predetermined impact force of 70 kdynes (severe injury). Sham-operated animals underwent all surgical procedures, including laminectomy, but their spinal cords were not injured. After surgery, animals were housed separately and treated with subcutaneous lactated Ringer's solution to prevent dehydration. Manual bladder expression was performed twice daily. Prophylactic antibiotic gentamicin was administered daily for 7 days to prevent urinary tract infections. Animal tissue was harvested 4-weeks post SCI, and either perfused and stored at 4°C, or snap-frozen in liquid nitrogen and stored at −80**°**C until the time of assay.

### Perfusion fixation

4-weeks post-SCI, animals were anesthetized as described above, then received an intracardial injection of heparin (0.1 cc) and perfused transcardially with physiological saline, followed by 100 ml of 4% (v/v) PFA (paraformaldehyde) in PBS. The brains were removed and placed in 4% PFA at 4°C overnight, then transferred to 20% (w/v) sucrose in 0.1 M PBS until sectioned.

### Immunohistochemistry

Animals were perfused with 4% PFA solution as described above, and brains were processed for cryostat sectioning. Serial coronal sections (50 μm) (−0.5 to −2.4 mm Bregma) (Hof, 2000) were stored free-floating in cryostat media (30% ethylene glycol, 30% sucrose, 0.1 M PBS, pH 7.4) at −20**°**C then rinsed with 0.1 M PBS (pH 7.4). Tissue sections were blocked/permeabilized by treatment with 5% normal goat serum and 0.4% (v/v) Triton X-100. Sections were incubated for 48 h at 4**°**C with POMC or NPY, and NeuN primary antibodies (1:200). Primary antibody binding was detected with Alexa Fluor secondary antibody conjugates (1:500). Controls lacking the primary antibody were run in parallel. Sections were counterstained with DAPI and coverslipped with Vectashield mounting medium for confocal analysis.

### Protein extraction and immunoblot analysis

Mice heart and pancreas tissue were harvested and homogenized in a Dounce homogenizer with extraction/lysis buffer (w/v) (20 mM Tris/HCl, pH: 7.5, 150 mM NaCl, 1% Triton X-100; 1 mM EDTA, 1 mM EGTA, 2.5 mM pyrophosphate, 1 mM 2-glycerophosphate) containing protease and phosphatase inhibitor cocktails and then centrifuged at 15 300 ***g*** for 2 min. VAT was harvested and homogenized in a Dounce homogenizer with extraction/lysis buffer (w/v) (50 mM Tris/HCl, pH: 7.4; 150 mM NaCl; 1% Triton X-100; 1% (v/v) Nonidet P40, 0.1% (w/v) SDS) containing protease and phosphatase inhibitor cocktails and then centrifuged at 15 300 ***g*** for 5 min. Lysates were mixed with 2× Laemmli loading buffer. Equal amounts of protein were resolved on 10–20% gradient Tris/HCl pre-casted gels, to separate proteins with a wide range of molecular masses, transferred to PVDF membranes and placed in blocking buffer (0.1% Tween-20, 0.4% I-block in PBS) overnight. Membranes were then incubated with primary antibodies followed by the appropriate HRP (horseradish peroxidase)-conjugated secondary antibody (1:1000). Visualization of the signal was enhanced by chemiluminescence using a Phototope-HRP detection kit. Quantification of bands corresponding to changes in protein levels was made using scanned densitometric analysis and NIH Image Program 1.62f, and normalized to β-Actin, JAK2^Total^, STAT3^Total^, p38^Total^ MAPK or RhoA where appropriate. Between group differences in immunoblots were analyzed using one-way ANOVA, followed by Tukey *post hoc* comparison and reflect percent change from naive control animals. Data are expressed as means ± S.E.M. A significance level of *P*≤0.05 was accepted as different from control.

### RhoA activation assay

Heart tissue protein lysate was prepared as described above, and activated RhoA was selectively assayed using Cell Biolabs, Inc. RhoA Activation Assay Kit according to the manufacturer's instructions. Briefly, 500 μl of sample was brought to a 1 ml volume using 1× Assay lysis buffer (125 mM HEPES, pH 7.5, 750 mM NaCl, 5% (v/v) Nonidet P40, 50 mM MgCl_2_, 5 mM EDTA, 10% (v/v) glycerol). 40 μl of resuspended Rhotekin RBD (Rho-binding domain) agarose bead slurry was added and incubated at 4°C for 1 h with gentle agitation. The beads were pelleted by centrifugation at 14 000 ***g*** for 10 s, and the supernatant was aspirated and discarded. The pelleted beads were washed three times in 500 μl of 1× Assay lysis buffer (described above), resuspended in 2× Laemmli loading buffer and boiled (98°C) for 5 min. Beads were carefully discarded. Remaining immunoprecipitates were separated on 10–20% (w/v) Tris/HCl pre-casted gels and analyzed by immunoblotting using mouse monoclonal anti-RhoA antibody and HRP-conjugated mouse secondary antibody. Partially purified recombinant RhoA and non-hydrolizable GTPγS were run as positive controls, and GDP was run as a negative control.

### ROCK activity assay

Heart tissue protein lysate was prepared as described above and analyzed for ROCK activity using Cell Biolabs, Inc. ROCK Activity Immunoblot Kit according to the manufacturers’ instructions. Briefly, 25 μl of sample was mixed with 50 μl of 1× kinase [250 mM Tris, pH 7.5, 100 mM MgCl2, 50 mM glycerol-2-phosphate, 1 mM Na3VO4)/ATP (10 mM)/ROCK substrate (0.25 mg/ml recombinant MYPT1 (myosin phosphatase target subunit 1)] and incubated at 30°C for 1 h with gentle agitation. The kinase reaction was stopped by resuspension in 25 μl of 4× Laemmli loading buffer. Samples were boiled (98°C) for 5 min and centrifuged at 12 000 ***g*** for 10 s. Supernatants were analyzed by immunoblotting using rabbit polyclonal anti-phospho-MYPT1^Thr696^ antibody and HRP-conjugated rabbit secondary antibody. Active ROCKII (10 ng active ROCK-II in 25 mM Tris, pH 7.5, 10 mM MgCl2, 5 mM glycerol-2-phosphate, 0.1 mM Na3VO4, 10% (v/v) glycerol, 0.1% (w/v) BSA) was run as a control.

### Co-immunoprecipitation

VAT and pancreas protein lysate were prepared as described above. Seventy microliters of Trueblot™ anti-mouse or anti-rabbit IgG immunoprecipitation beads were added to 200 μg of sample, and the mixture was rotated at 4°C for 2 h in a microcentrifuge tube for preclearing. The beads were pelleted by centrifugation at 15 300 ***g*** for 30 s. The supernatant was recovered and mixed with either 5 μg/ml of anti-NLRP3 or 5 μg/ml anti-ASC [apoptosis-associated speck-like protein containing a caspase recruitment domain (CARD, q.v.)] primary antibody and incubated at 4°C overnight. Seventy microliters of anti-mouse or anti-rabbit IgG beads was added and incubated for 2 h and then centrifuged at 15300 ***g*** for 30 s, and the supernatant was aspirated and discarded. The pelleted beads were washed six times in extraction/lysis buffer (described above), resuspended in 2× Laemmli loading buffer, and heated at 95°C for 3 min. Beads were carefully discarded. Remaining immunoprecipitates were separated on 10–20% (w/v) Tris/HCl pre-casted gels and analyzed by immunoblotting using the appropriate antibodies and HRP-conjugated mouse IgG Trueblot™ or rabbit IgG Trueblot™ secondary antibodies. Normal serum was run as a control.

## RESULTS

### POMC and NPY expression levels in the hypothalamus are altered following chronic SCI

**Figure 1:**
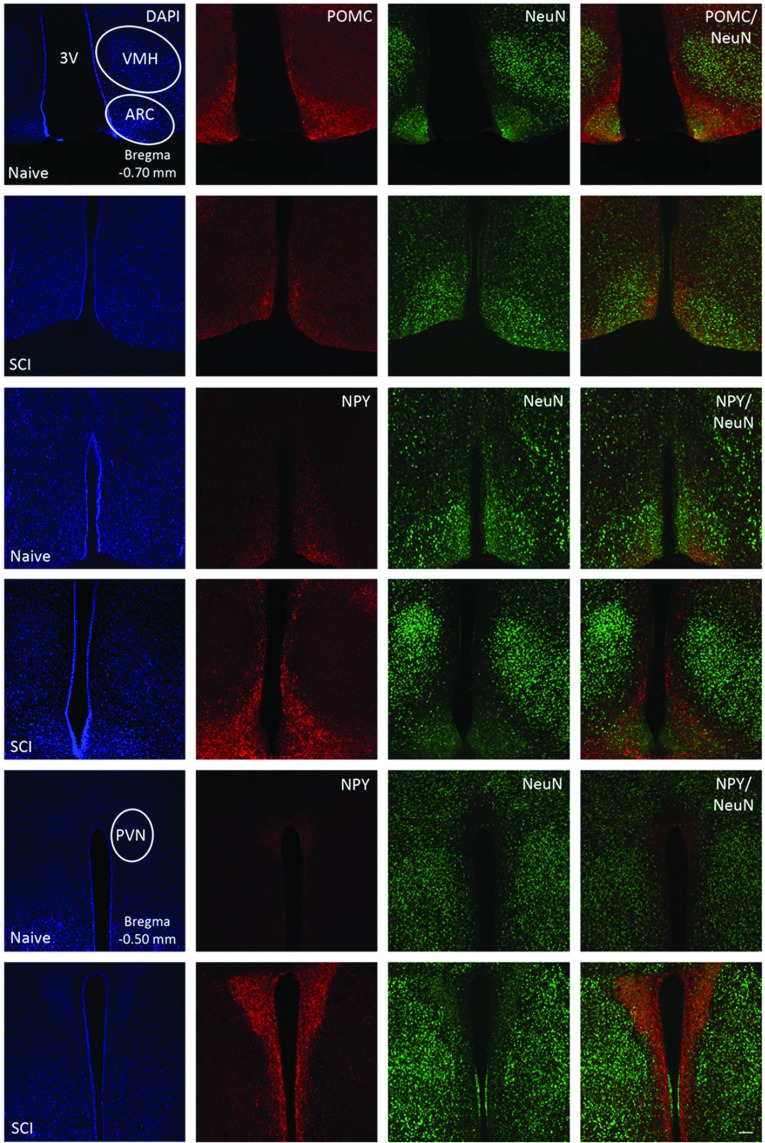
Confocal images of POMC and NPY localization and expression in hypothalamic ARC and PVN neurons in control and SCI mice Mouse brain coronal sections (−0.5 to −2.4 mm Bregma) were immunostained with POMC or NPY (Red), and the neuronal marker NeuN (Green) and counterstained using DAPI (Blue). In naïve mice, brain regions corresponding to the ARC (Row 1, Blue) are positively immunostained with POMC (Row 1, Red) and NeuN (Row 1, Green) and POMC is localized in subpopulations of NeuN positive (Row 1, Merged) positive cells. SCI brains (1-month post injury) (Row 2) have substantially reduced POMC (Row 2, Red) immunoreactivity in ARC neurons compared to naïve control. Also in naïve mice, low levels of ARC neurons are positively immunostained with NPY (Row 3, Red) and NeuN (Row 3, Green), however 1-month post-SCI, there is substantially increased NPY (Row 4, Red) immunoreactivity when compared to naïve control. In addition, in naïve mice, brain regions corresponding to the PVN (Row 5, Blue) display low levels of NPY immunoreactivity (Row 5, Red), as well as NeuN positive immunostaining (Row 5, Green). SCI brains (1-month post injury) (Row 6) have substantially increased NPY (Row 6, Red) immunoreactivity in PVN neurons compared to naïve control. Scale Bars=50 μM. Visual representation of analysis for all animals (*n*=8).

It is well established that POMC and NPY gene expression and peptide levels are abundantly produced in the ARC and have targeted innervation including PVN (paraventricular nucleus) melanocortin neurons involved in energy balance and metabolic homeostasis (Cone, 2005; Smart et al., 2006; Myers and Olson, 2012). Confocal images (Figure 1) illustrate the regional distribution and cell type expression of POMC and NPY. Here we show that subpopulation of hypothalamic ARC neurons are positively immunostained with POMC (red) and the neuronal marker NeuN (green) (Row 1) in the naïve control. Following chronic SCI, ARC neurons show substantially less intense POMC (red) immunoreactivity (Row 2) compared to naïve control. We also observe that subpopulations of hypothalamic ARC neurons are positively immunostained with NPY (red) and NeuN (green) (Row 3) and that following chronic SCI, ARC neurons show substantially more NPY (red) immunoreactivity (Row 4) compared to naïve control. Within the PVN, NPY (red) immunostaining is evident in dorso-medial regions in naïve control (Row 5), and following SCI, there is substantially greater NPY (red) immunoreactivity throughout the entire PVN in subpopulations of NeuN (green) positive neurons (Row 6) compared to naïve control. Thus, these data support our previous report (Bigford et al., 2012) illustrating altered expression levels of hypothalamic signaling peptides, and extend evidence for dysregulated neuroendocrine and melanocortin system signaling following SCI.

### Chronic SCI induces a significant increase in heart leptin receptor protein expression, JAK2/STAT3/p38 MAPK signaling and concurrent RhoA/ROCK activation

**Figure 2:**
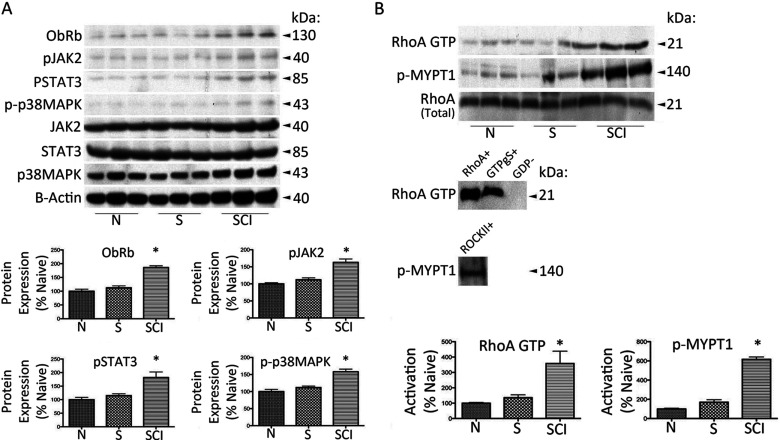
Immunoblot analysis of leptin receptor, JAK/STAT/p38 MAPK signaling, and RhoA/ROCK activation in heart from control and SCI mice (**A**) Long form leptin receptor (Ob-Rb) expression is significantly increased 1-month post SCI when compared to the naïve (N) and sham-operated (S) control. Similarly, JAK2 phosphorylation, STAT3 phosphorylation, and p38 MAPK phosphorylation are all significantly increased in SCI when compared to the naïve (N) and sham-operated (S) control. (**B**) Additionally, the activation of RhoA (RhoA GTP) and the activation of the ROCK substrate p-MYPT1 are both significantly elevated 1-month post-SCI when compared to the naïve (N) and sham-operated (S) control. Partially recombinant RhoA (RhoA +) and non-hydrolizable GTPγS (GTPγS +) were used as positive controls for RhoA the assay. GDP (GDP−) was used as a negative control. Active ROCK (ROCKII+) was used as a positive control for the ROCK assay. JAK2^Total^, STAT3^Total^ and p38 MAPK^Total^ were used as internal standards. β-actin was used as a protein loading control. Statistics are according to data analysis methods described. *P*≤0.05. *n*=8 for each group.

Various leptin induced signaling mechanisms have been implicated in cardiovascular pathology. Therefore, we next examined whether chronic SCI affects signaling in the heart associated with leptin mediated cardiac metabolic dysfunction (Figure 2). We observe that the long form of the leptin receptor (Ob-Rb) protein expression in the heart is significantly increased following chronic SCI when compared to naïve and sham-operated control (Figure 2A). Similarly, JAK2 tyrosine phosphorylation (JAK2^P^), STAT3 tyrosine phosphorylation (STAT3^P^), and p38 MAPK threonine and tyrosine phosphorylation (p38^P^) are significantly increased following SCI compared to naïve and sham-operated control (Figure 2A). Additionally, RhoA activation (RhoA-GTP) in heart tissue was examined by precipitation with its endogenous substrate Rhotekin (Figure 2B). In its active state, RhoA-GTP binds to the RBD of Rhotekin, initiating downstream signaling cascades and regulating a variety of biological responses. Here we used Rhotekin RBD agarose beads to selectively isolate and pull-down RhoA-GTP from tissue lysates. Chronic SCI induced a significant increase in RhoA-GTP compared to naïve and sham-operated control. Partially recombinant *RhoA* was used to accurately identify RhoA immunoblots. Non-hydrolizable GTPγS loaded samples resulted in activation and precipitation of RhoA, whereas GDP-loaded samples did not activate or precipitate RhoA, functioning as positive and negative controls, respectively. Further, RhoA effects are mediated via its downstream effector ROCK. Activated ROCK mediates RhoA signaling through the phosphorylation of several substrates. Specifically, activated ROCK functions to inactivate myosin phosphatase through the phosphorylation of MYPT1 at Thr^696^, conferring cytoskeletal changes observed in leptin-induced cardiomyocyte pathologies. Heart tissue ROCK activation was examined by incubation with its recombinant physiological substrate MYPT1, and assayed for the activation of MYPT1^Thr696^ (p-MYPT1) (Figure 2B). Consistent with our RhoA data, chronic SCI induced a significant increase in MYPT1 threonine phosphorylation (activated) compared to naïve and sham-operated control. Active ROCKII was used as a positive control, activating MYPT1 and accurately identify threonine phosphorylated MYPT1 immunoblots. Taken together, these data provide evidence that chronic SCI incites several signaling mechanisms that are associated with leptin-mediated metabolic dysfunction in the heart, and may contribute to pathophysiology associated with CVD.

### Chronic SCI results in the formation of the NLRP3 inflammasome multi-protein complex in VAT and pancreas

**Figure 3:**
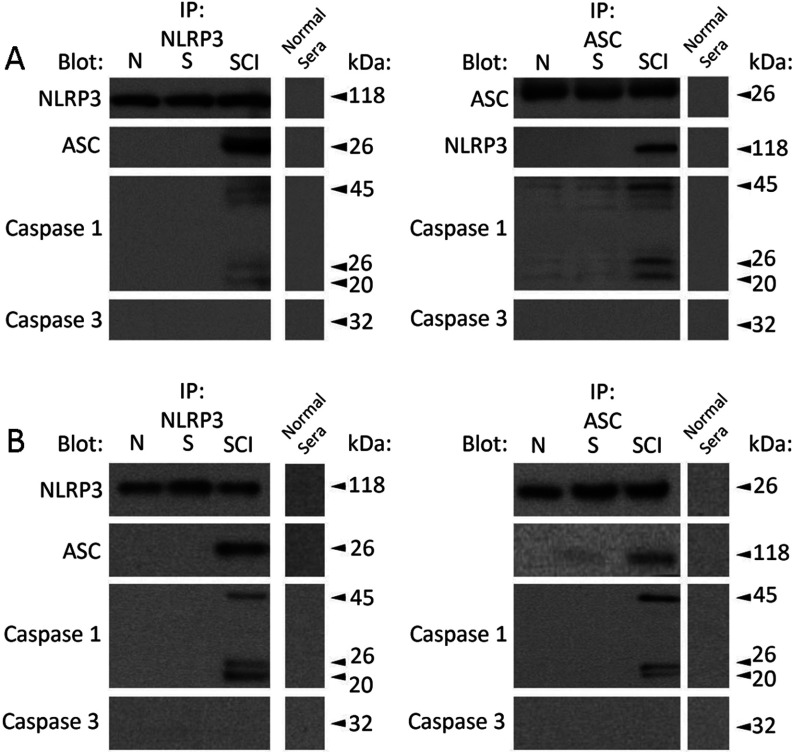
NLRP3, ASC, and caspase-1 form the *NLRP3 inflammasome* multi-protein complex in VAT and pancreas in SCI mice (**A**) Co- IP (immunoprecipitation) from VAT using NLRP3 antisera precipitated its cognate protein, ASC and multiple variants of caspase-1 (pro-forms 45, 50 kDa, cleaved-forms 20, 26 kDa) 1-month post-SCI but not in the naïve (N) or sham-operated (S) control. Similarly, reciprocal IP using ASC antisera precipitated its cognate protein, as well as NLRP3 and pro- and cleaved- forms of caspase-1. Caspase-3 was not immunoprecipitated using either NLRP3 or ASC, and normal serum did not immunoprecipitate any inflammasome-associated proteins, illustrating specificity in protein interactions and serving as a negative control, respectively. (**B**) IP from pancreas using NLRP3 antisera precipitated its cognate protein, ASC and multiple variants of caspase-1 (pro-forms 45, 50 kDa, cleaved-forms 20, 26 kDa) 1-month post-SCI but not in the naïve (N) or sham-operated (S) control. Similarly, reciprocal IP using ASC antisera precipitated its cognate protein, as well as NLRP3 and pro- and cleaved- forms of caspase-1. Caspase-3 was not immunoprecipitated using either NLRP3 or ASC, and normal serum did not immunoprecipitate any inflammasome-associated proteins, illustrating specificity in protein interactions and serving as a negative control, respectively. Representative analysis for all animals (*n*=8).

The NLRP3 inflammasome has been shown to contribute to inflammatory processes, metabolic dysfunction and diabetes risk (Schroder et al., 2010; Strowig et al., 2012; Wen et al., 2012). To characterize the interaction of inflammasome proteins in naïve and sham-operated control, and compare them with protein interactions accompanying chronic SCI, we performed co-immunoprecipitation of VAT and pancreatic lysate using either anti-NLRP3 or anti-ASC antibody (Figure 3). Figure 3(A) shows that co-immunoprecipitation from VAT using either anti-NLP3 or -ASC antisera formed reciprocal protein–protein interactions, as well as interactions with the pro- and cleaved- forms of caspase-1 following chronic SCI. However, these interactions were not observed in naïve or sham-operated control. Caspase-3 was not immunoprecipitated under these experimental conditions, and normal serum did not immunoprecipitate any inflammasome-associated proteins, illustrating specificity in protein interactions and serving as a negative control, respectively. Similarly, co-immunoprecipitation from pancreatic tissue (Figure 3B) using either anti-NLRP3 or -ASC antisera resulted in reciprocal protein–protein interactions as well as interactions with the pro- and cleaved- forms of caspase-1 following chronic SCI. These interactions were not observed in naïve or sham-operated control. As with VAT, caspase-3 was not immunoprecipitated under these experimental conditions, and normal serum did not immunoprecipitate any inflammasome-associated proteins, again illustrating specificity in protein interactions and serving as a negative control, respectively. Normalization of co-immunoprecipitants to precipitated (target) protein was performed as an estimation of variability and additional control (Supplementary Figure S1 available at http://www.asnneuro.org/an/005/an005e121add.htm). These data provide the first direct biochemical evidence for the formation of the NLRP3 inflammasome in VAT and pancreas following chronic SCI.

### Chronic SCI results in a significant increase in the proteolytic cleavage and activation of caspase-1, IL-1β, and IL-18 in VAT and pancreas

**Figure 4:**
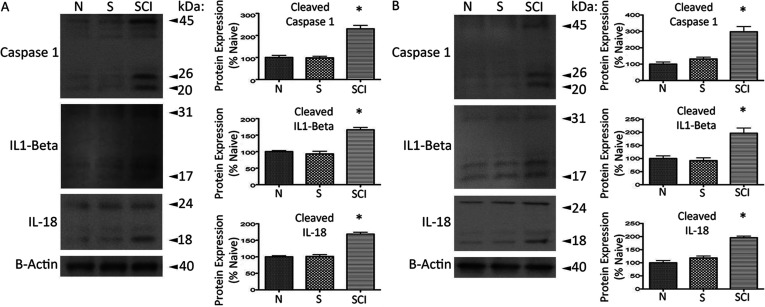
Immunoblot analysis of proteolytic processing and activation of caspase-1, IL-1β, and IL-18 in VAT and pancreas in control and SCI mice (**A**) In VAT there is a significant increase in proteolytic cleavage of pro-caspase 1 (45, 50 kDa) to enzymatically active forms (20, 26 kDa) 1-month post-SCI compared to the naïve (N) and sham-operated (S) control. There is also a significant increase in proteolytic cleavage of IL-1β (31 kDa) and IL-18 (24 kDa) to their enzymatically active forms (17, 18 kDa, respectively) 1-month post-SCI when compared to the naïve (N) and shame-operated (S) control. (**B**) In VAT there is a significant increase in proteolytic cleavage of pro-caspase 1 (45, 50 kDa) to enzymatically active forms (20, 26 kDa) 1-month post-SCI compared to the naïve (N) and sham-operated (S) control. There is also a significant increase in proteolytic cleavage of IL-1β (31 kDa) and IL-18 (24 kDa) to enzymatically active forms (17, 18 kDa, respectively) 1-month post-SCI when compared to the naïve (N) and shame-operated (S) control. β-actin was used as a protein loading control. Statistics are according to data analysis methods described. *P*≤0.05. *n*=8 for each group.

NLRP3 inflammasome formation promotes the proximity-induced autocatalytic activation of caspase-1 and mediates the cleavage/activation and secretion/release of the pro-inflammatory cytokines IL-1β and IL-18 implicated in several metabolic disorders (Schroder et al., 2010; Strowig et al., 2012; Wen et al., 2012). To determine whether chronic SCI induces processing of caspase-1 and the pro-inflammatory cytokines IL-1β and IL-18, we analyzed VAT and pancreatic lysate, comparing chronic SCI, naïve and sham-operated control (Figure 4). In VAT, chronic SCI resulted in a significant increase in the proteolytic cleavage products of caspase-1 (20, 26 kDa), IL-1β (17 kDa) and IL-18 (18 kDa) when compared to naïve and sham-operated control (Figure 4A). Correspondingly, in pancreatic lysate chronic SCI also induced a significant increase in the proteolytic cleavage products of caspase-1, IL-1β, and IL-18 (Figure 4B). These results demonstrate that chronic SCI initiates the coalescing of the NLRP3 molecular platform leading to the activation of caspase-1, IL-1β, and IL-18 in VAT and pancreas. This supports the involvement of NLRP3 inflammasome-associated metabolic dysfunction following chronic SCI.

## DISCUSSION

We report that chronic SCI results in altered expression of POMC and NPY, key regulators of metabolic energy balance within the hypothalamic ARC and PVN. In heart tissue, we observe a significant increase in leptin receptor (Ob-Rb) expression, and downstream activation of JAK2/STAT3, p38 MAPK, and RhoA/ROCK signaling following chronic SCI, cited in cardiac dysfunction and inflammation related to CVD progression. Furthermore, we identify the formation of the NLRP3 inflammasome and subsequent activation of caspse-1, IL-1β, and IL-18 in both VAT and pancreas, implicated in metabolic dysfunction and autoinflammatory diseases. Taken together, these support our previous findings illustrating centrally mediated inflammatory processes, and provide the direct evidence for peripheral inflammation associated with CVD risk and diabetes in chronic SCI.

There is emerging evidence for centrally derived leptin signaling dysfunction related to metabolic energy balance following chronic SCI. Leptin effects through distinct ARC neurons activate the transcription of POMC. POMC gene deletion in rodents results in significantly reduced metabolic rate, hyperphagia and obesity, primarily from altered lipid metabolism (Yaswen et al., 1999; Challis et al., 2004), whereas neuronal overexpression of POMC has been shown to reduce food intake and attenuate obesity in *ob/ob* mice and obese Zucker rats (Li et al., 2003; Mizuno et al., 2003). Conversely, leptin signals in a second distinct subpopulation of ARC neurons expressing the potent orexigenic peptide NPY. NPY release is correlated with food intake (Kalra et al., 1991), and NPY-ergic *tone* mediates hyperphagia (Stanley et al., 1986; Zarjevski et al., 1993; Sainsbury et al., 1997; Raposinho et al., 2001), decreased energy expenditure (Billington et al., 1991), and induces direct neuroendocrine/metabolic effects conferring significant increases in energy storage, excessive fat and weight gain (Zhang et al., 2011). Furthermore, NPY receptors expressed on POMC neurons in the ARC confer NPY-mediated inhibition/suppression of the primary anorexigenic system (Galas et al., 2002; Roseberry et al., 2004), and in this manner, produce inhibitory effects on POMC neurons of the primary melanocortin system. Therefore NPY orexigenic effects in the hypothalamus include both independent/direct signaling mechanisms and inhibitory regulation of anorectic signals.

As we previously reported, there is evidence of significantly attenuated hypothalamic leptin signaling and acquired leptin resistance following chronic SCI (Bigford et al., 2012). Here, we extend downstream leptin-mediated signal dysregulation, illustrating a decrease in post-SCI POMC protein expression within the ARC, and a substantial increase in NPY expression in the PVN. These observations support that dysregulated hypothalamic leptin signaling following chronic SCI attenuates both the downstream production of POMC and anorexigenic neuroendocrine pathways associated with the primary melanocortin system, and in parallel, NPY-ergic inhibition, resulting in exacerbated orexigenic effects. Further experiments identifying other known and important signal intermediates will continue to clarify SCI-induced pathology related to metabolic dysfunction.

There is additional evidence for leptin-mediated metabolic dysfunction in the heart. *In vitro*, leptin induces cardiomyocyte hypertrophy (Xu et al., 2004; Zeidan et al., 2011) involving MAPK and RhoA-GTP (Madani et al., 2006; Zeidan et al., 2006; Zeidan et al., 2008), which contribute to morphological and phenotypic changes in cardiac size, structure, function, and heart failure (Sweeney, 2010). Moreover, leptin has been shown to stimulate FAO and oxygen consumption in the heart (Atkinson et al., 2002) where imbalance in metabolic substrate utilization leads to cardiac dysfunction (Hou and Luo, 2011) and overall cardiac deficiency (Abel et al., 2008). This effect is shown to develop through JAK2/STAT3/p38 MAPK pathways (Nickola et al., 2000; Wold et al., 2002; Sharma et al., 2009; Lopaschuk et al., 2010), and interestingly, RhoA/ROCK-mediated cardiac hypertrophy is p38 MAPK-dependent (Zeidan et al., 2006; Zeidan et al., 2008), intimating p38 MAPK as a critical pathway involved in dysregulated metabolic and hypertrophic mechanisms. In fact, p38 activation is an intermediary of pro-inflammatory-induced tissue damage and the pathophysiology of heart disease (Kaiser et al., 2004; Kerkela and Force, 2006; Clark et al., 2007) and has been the target of therapeutic intervention strategies (Marber et al., 2010). Similarly, ROCK involvement in CVD comorbidities including hypertension (Mukai et al., 2001; Seko et al., 2003; Moriki et al., 2004), atherosclerosis (Rekhter et al., 2007; Mori-Kawabe et al., 2009; Wu et al., 2009) and cardiac hypertrophy and heart failure (Higashi et al., 2003; Satoh et al., 2003; Fukui et al., 2008; Phrommintikul et al., 2008), has been the focus of directed therapeutics. These efforts highlight the significance of these signaling pathways in CVD progression. Our data provide evidence that pathological processes involved with chronic SCI result in significant dysfunction in cardiac leptin signaling pathways. We find a significant increase in Ob-Rb expression in the heart, which is in contrary to Ob-Rb down-regulation previously observed in the ARC. Previous literature posits the concept of *selective* leptin resistance (Mark et al., 2002) where sympathoexcitatory actions of leptin are preserved, while central anorectic effects of leptin are impaired. This may explain, in part, how leptin contributes to hypertension and pro-atherogenic effects discussed above; however, the biological evidence for this remains unknown. One possibility is that cardiac tissue express several leptin receptors isoforms (Lollmann et al., 1997; Wold et al., 2002) that may confer distinct functional roles. This diversity is not observed in ARC neurons, which predominantly express Ob-Rb (Fei et al., 1997; Oswal and Yeo, 2010). In ARC neurons hyper-activation may lead to receptor down-regulation, attenuation of central signals and subsequent resistance, whereas in the heart, leptin may activate signaling through various receptor isoforms, and disperse modulatory and/or *feedforward* stimulation for up-regulated signaling intermediates.

Several other peripheral tissues are responsible for inflammation and metabolic dysfunction in SCI. Previous evidence supports that the total fat mass is significantly higher in SCI compared to able bodied, age-compared control (Maggioni et al., 2003; Spungen et al., 2003), with more recent reports illustrating that individuals with SCI have greater VAT cross-sectional area, associated with maladaptive metabolic profile, and predictive of impaired glucose tolerance, insulin resistance, and dyslipidemia (Edwards et al., 2008; Gorgey et al., 2011). A growing literature has demonstrated that the formation of a multi-protein NLRP3 inflammasome signaling complex and subsequent cytokine activation is an underlying mechanism of VAT (Vandanmagsar et al., 2011) and pancreatic (Schroder et al., 2010; Wen et al., 2012) inflammation contributing to metabolic dysfunction and diabetes. NLRP3 inflammasome formation induces the autocatalytic cleavage and activation of pro-caspase 1, in turn mediating the cleavage and activation of IL-1β and IL-18 (Schroder and Tschopp, 2010; Barker et al., 2011), experimentally and clinically linked to the development of metabolic pathologies (Netea et al., 2006). With increased adiposity, NLRP3 inflammasome activation leads to IL-1β-mediated insulin signal inhibition and induced TNFα (tumor necrosis factor) production, an established insulin resistance promoting cytokine (Vandanmagsar et al., 2011; Strowig et al., 2012). Similarly, NLRP3 inflammasome activation in pancreatic β-cells is triggered by hyperglycemia, and subsequent IL-1β production contributes to β-cell death, suggesting the NLRP3 inflammasome as a sensor of chronically elevated glucose and a mediator of pancreatic dysfunction (Dinarello et al., 2010; Strowig et al., 2012). Deficiency in any NLRP3 inflammasome component (*NLRP3^−/−^*, *ASC^−/−^*, *Casp1^−/−^*) has shown to be protective against the development of high-fat diet-induced obesity and improves glucose homeostasis in rodent models (Stienstra et al., 2010; Zhou et al., 2010; Stienstra et al., 2011; Vandanmagsar et al., 2011), and protective against chronic obesity-induced pancreatic damage (Youm et al., 2011). These data establish NLRP3 inflammasome activation as an intermediary of metabolic diseases. Correspondingly, several studies have shown that IL-1β and IL-18 play an important role in atherosclerotic lesions (Mallat et al., 2001; Kirii et al., 2003; Garg, 2011); however, recent work investigating the NLRP3 inflammasome within the lesion has yet to be clearly established.

Importantly, recent literature has brought attention to inflammasome activation induced pathomechanisms in SCI. The NLRP1 inflammasome–having similar components and output to NLRP3–is triggered acutely following SCI within the injury epi-center and contributes to inflammation related lesion volume and functional deficits (de Rivero Vaccari et al., 2008). These observations extend to focal injuries in traumatic brain injury and ischemic stroke (Abulafia et al., 2009; de Rivero Vaccari et al., 2009) and suggest inflammasome activation as an acute innate response to trauma in the CNS. Our data extend that following chronic SCI, NLRP3 inflammasome formation, and activation of caspase-1, IL-1β, and IL-18 is actuated in peripheral tissues such as VAT and pancreas. We provide biological affirmation of this established marker of chronically acquired low-grade inflammation associated with metabolic disorders and component CVD risk. Taken together, these data illustrate that the inflammasome component of the innate immune response, are eminent in acute-phase adaptation to CNS perturbations, as well as long-term systemic pathologies secondary to injury.

**Figure 5:**
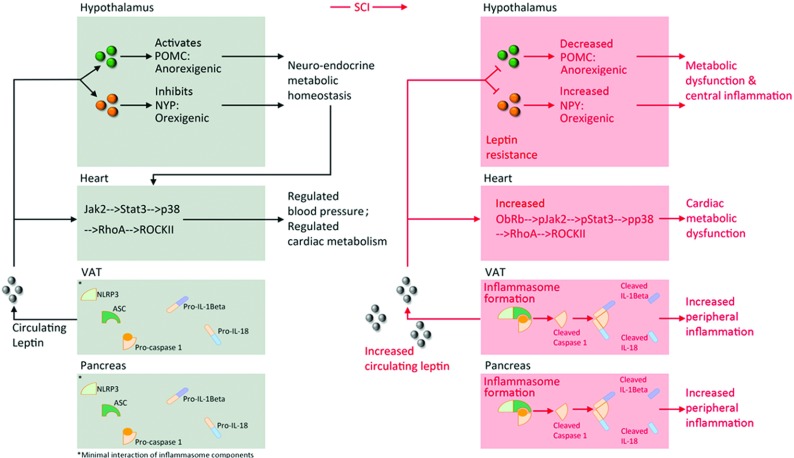
Model of observed central and peripheral metabolic dysfunction and inflammatory processes following chronic SCI Chronic SCI results in leptin resistance and subsequent attenuation of POMC expression in the ARC and enhanced NPY expression in the PVN, promoting imbalanced metabolic homeostasis. Leptin resistance is an established surrogate for chronic central inflammation. Also, following chronic SCI there is increased cardiac leptin observed (Ob-Rb-mediated), activation of JAK2/STAT3/p38 MAPK and RhoA/ROCK pathways, associated with hypertension, hypertrophy and dysregulated cardiac metabolism. Finally, chronic SCI-induced VAT and pancreatic NLRP3 inflammasome formation and activation of caspase-1, IL-1β, and IL-18. These observations in heart, VAT, and pancreas are associated with chronic peripheral/systemic inflammatory processes.

Here we provide evidence to support hypothalamic neuroendocrine dysfunction associated with chronic inflammation, cardiac metabolic dysfunction associated with CVD progression, and VAT and pancreatic markers of inflammation and metabolic disease following chronic SCI (Summarized, Figure 5). These findings develop our understanding of underlying mechanisms that contribute to component cardiometabolic disease risk. As we begin to view SCI as an inciting event for chronically acquired CVD and cardiometabolic disease risk, we can direct both therapeutic and rehabilitation countermeasures, and investigate appropriate interventions that may attenuate these pathologies and improve quality of life.

## Online data

Supplementary data
