# Supplementary material for: Neuroendocrine and cardiac metabolic dysfunction and NLRP3 inflammasome activation in adipose tissue and pancreas following chronic spinal cord injury in the mouse
Source: ASN Neuro. 2013 Sep 4;5(4):e00121. doi: 10.1042/AN20130021 (PMC3789215; doi:10.1042/AN20130021)
Supplement: Supplementary data [file an005e121add.pdf]

ASN NEURO RESEARCH ARTICLE

ASN NEURO 5(4):art:e00121.doi:10.1042/AN20130021

OPEN ACCESS

Neuroendocrine and cardiac metabolic dysfunction and NLRP3 inflammasome activation in adipose tissue and pancreas following chronic spinal cord injury in the mouse

Gregory E. Bigford*[1], Valerie C. Bracchi-Ricard*, Robert W. Keane†, Mark S. Nash*‡§ and John R. Bethea*‡||

*The Miami Project to Cure Paralysis, University of Miami Miller School of Medicine, Miami, FL, U.S.A.

†Department of Physiology, University of Miami Miller School of Medicine, Miami, FL, U.S.A

‡Department of Neurological Surgery, University of Miami Miller School of Medicine, Miami, FL, U.S.A.

§Department of Rehabilitation Medicine, University of Miami Miller School of Medicine, Miami, FL, U.S.A.

||Department of Microbiology and Immunology, University of Miami Miller School of Medicine, Miami, FL, U.S.A.

SUPPLEMENTARY DATA

Supplementary Figure S1 is on the following page.

[1]To whom correspondence should be addressed (email gbigford@med.miami.edu).

© 2013 The Author(s) This is an Open Access article distributed under the terms of the Creative Commons Attribution Licence (CC-BY) (http://creativecommons.org/licenses/by/3.0/) which permits unrestricted use, distribution and reproduction in any medium, provided the original work is properly cited.

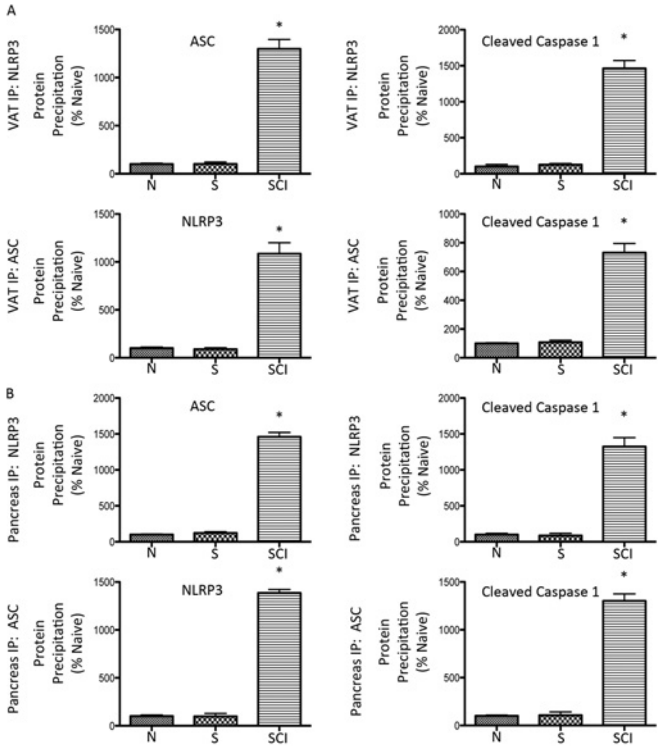

Figure S1 Quantification of NRLP3 inflammasome components from VAT and pancreas co-immunoprecipitation in control and SCI mice
(**A**) In VAT IPs using NLRP3 antisera, there is significantly greater ASC, and cleaved caspase 1 immunoprecipitants 1-month post-SCI when compared to naïve (N) and sham-operated (S) control. Similarly, in VAT IP's using ASC antisera, there is significantly greater NLRP3, and cleaved caspase 1 immunoprecipitants 1-month post-SCI when compared to naïve (N) and sham-operated control. (**B**) In pancreas IP's using NLRP3 antisera, there is significantly greater ASC, and cleaved caspase 1 immunoprecipitants 1-month post-SCI when compared to naïve (N) and sham-operated (S) control. Similarly, in pancreas IPs using ASC antisera, there is significantly greater NLRP3, and cleaved caspase 1immunoprecipitants 1-month post-SCI when compared to naïve (N) and sham-operated (S) control. NLRP3 and ASC were used as internal standards. Statistics are according to data analysis methods described. $P \leqslant 0.05$. $n = 8$ for each group.

© 2013 The Author(s) This is an Open Access article distributed under the terms of the Creative Commons Attribution Licence (CC-BY) (http://creativecommons.org/licenses/by/3.0/) which permits unrestricted use, distribution and reproduction in any medium, provided the original work is properly cited.
